# Living in Temporary Ponds Loading Giant Genomes: The Neotropical Annual Killifish Genus *Austrolebias* as New Outstanding Evolutionary Model

**DOI:** 10.3389/fgene.2022.903683

**Published:** 2022-06-20

**Authors:** Graciela García, Verónica Gutiérrez, Néstor Ríos

**Affiliations:** Sección Genética Evolutiva, Facultad de Ciencias, UdelaR, Montevideo, Uruguay

**Keywords:** neotropical, killifish, *Austrolebias*, giant genomes, evolutionary model

## Abstract

The term Annual killifish describes a short-lived and amazing group of vertebrates inhabiting temporary ponds exposed to an extremely variable environment during its short lifespan in South America and Africa, leading to the death of the entire adult population during the dry season. *Austrolebias* is a specious genus of the family Rivulidae, with ∼58 currently recognized species, extensively distributed in the temperate Neotropical region. Herein, we reviewed different aspects of the evolutionary biology with emphasis on the genome dynamic linked to the burst speciation process in this genus. *Austrolebias* constitutes an excellent model to study the genomic evolutionary processes underlying speciation events, since all the species of this genus analyzed so far share an unusually large genome size, with an average DNA content of 5.95 ± 0.45 picograms per diploid cell (mean C-value of about 2.98 pg). The drastic nuclear DNA–increasing would be associated with a considerable proportion of transposable elements (TEs) found in the *Austrolebias* genomes. The genomic proportion of the moderately repetitive DNA in the *A. charrua* genome represents approximately twice (45%) the amount of the repetitive components of the highly related sympatric and syntopic rivulinae taxon *Cynopoecilus melanotaenia* (25%), as well as from other rivulids and actinopterygian fish. These events could explain the great genome instability, the high genetic diversity, chromosome variability, as well as the morphological diversity in species of *Austrolebias*. Thus, species of this genus represent new model systems linking different evolutionary processes: drastic genome increase, massive TEs genomic representation, high chromosome instability, occurrence of natural hybridization between sister species, and burst speciation events.

## Introduction

It has been proposed that model organisms are widely used in research as accessible and convenient systems to study a particular area or question in biology (Russell et al., 2017). In recent years they have accelerated the proliferation of experimental approaches, such as high-throughput sequencing, CRISPR gene editing, transgenesis, and other technologies which have enabled new insights, particularly when a trait of interest is most readily observed in a non-traditional model organism (Juntti, 2019).

**FIGURE 1:**
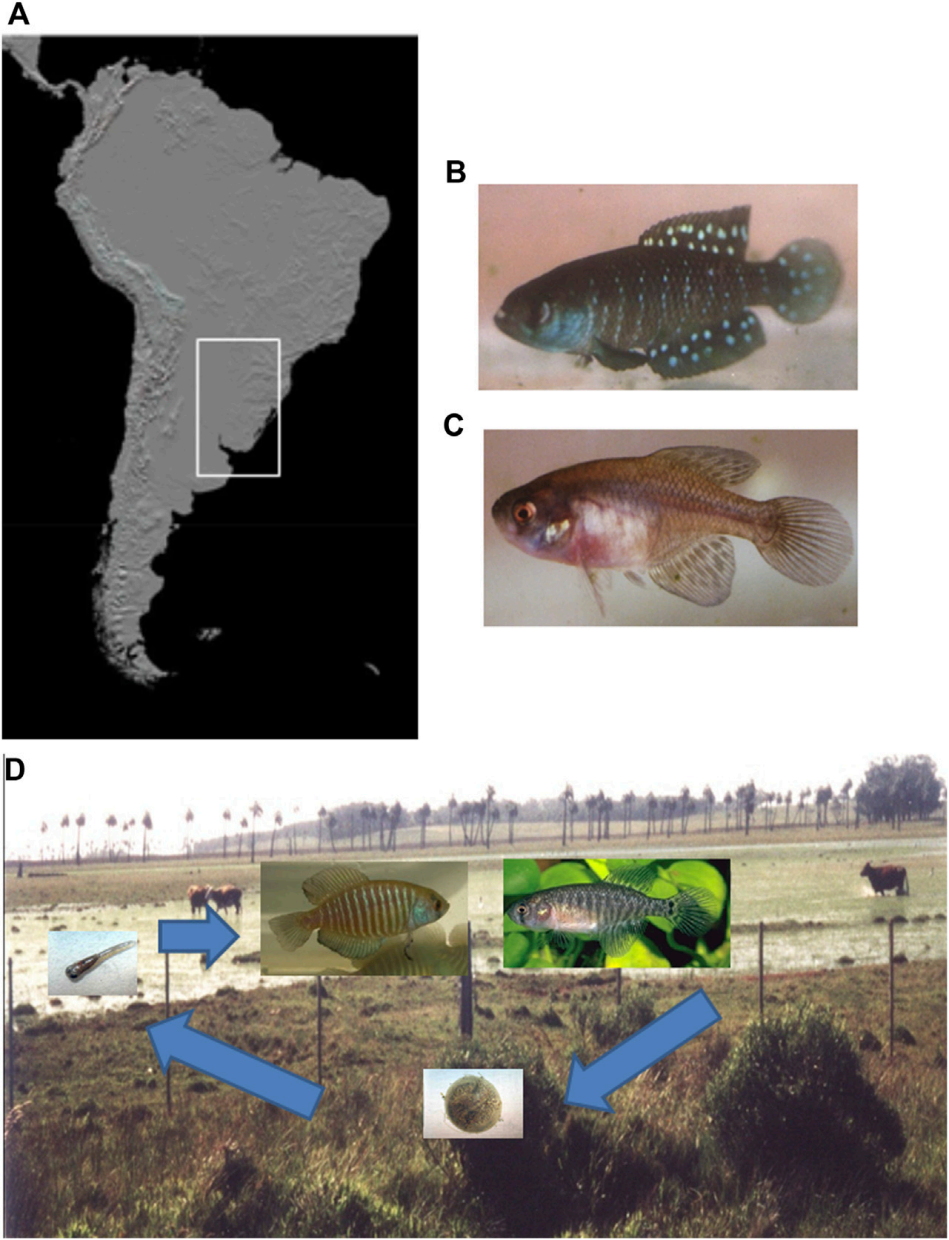
Continental occurrence area, specimens, and life cycle of annual fish *Austrolebias.* **(A)** Distribution area of the *Austrolebias* genus in South America (white rectangle). **(B)** Adult male of the *A. affinis* species group. **(C)** Adult female of the *A. affinis* species group. **(D)** Life cycle of annual fish *Austrolebias* in temporary ponds from the R.16, Rocha Department, in Uruguay. *A. charrua* male (left) and female (right) are shown, the embryos remain in a prehatching stage of developmental arrest and hatch in the next rainy season when the ponds are flooded. The resulting juveniles reach sexual maturity in a few weeks.

In this sense, annual fish have been focused on as an important model organism as a consequence of an evolutionary adaption to its extreme habitat (Berois et al., 2014; Reichard and Polačik, 2019). This fish group inhabiting temporary ponds in South America and Africa is a unique, short-lived, vertebrate model presenting developmental, ecological, physiological, genetics, and evolutionary peculiar adaptations (García et al., 2014; Loureiro and de Sá, 2016; Berois et al., 2016, Reichard and Polačik, 2019). In particular, *Austrolebias* is a specious genus of the family Rivulidae, with ∼58 currently recognized species (Froese and Pauly, 2021), extensively distributed in the temperate Neotropical region in two different South American basins (Figure 1A): La Plata-Paraná-Paraguay-Uruguay basin and Patos-Merin system (Costa, 2006; Loureiro et al., 2011; Volcan et al., 2014). Seasonal ponds inhabited by *Austrolebias* range from rounded to irregularly shaped shallow depressions in grasslands and seasonal flooded areas adjacent to rivers or large permanent wetlands (Loureiro et al., 2016). Seasonal patches strongly push individuals to accommodate spatially to optimize food supply, metabolic efficiency, mating opportunities, and predator avoidance, all in a few months (Wilbur, 1997). Therefore, they are freshwater teleosts exposed to an extremely variable environment during their short lifespan (Berois et al., 2016). During the rainy season the adults reproduce and generate desiccation-resistant embryos that remain buried in the muddy bottom of the dry ponds (Figures 1B–D). The embryos hatch in the next rainy season since the ponds are flooded, whereas the entire adult population dies during the dry season (Berois et al., 2016). The resulting juveniles reach sexual maturity in a few weeks, until a new reproductive cycle begins (Wourms, 1967; Arezo et al., 2005). Therefore, the survival of the species becomes entirely dependent upon buried embryos and this unique life cycle is correlated to the seasonal environments (Berois et al., 2016).

One of the strengths of using a model organism is the ability to dispose of all possible information, including different sources of data about the analyzed system, improving the interpretation of results. Currently, studies on the evolutionary biology (García et al., 2002, 2009, 2014, 2019, 2020), systematics and conservation (Loureiro et al., 2018), ethology (Passos et al., 2013), ecology (Arim et al., 2011), reproductive strategies (Arezo et al., 2005, 2014), regulation of developmental pathways (Berois et al., 2016), aging (Gutiérrez, 2014), and neurogenesis (Fernández et al., 2020) on several species of *Austrolebias* are in progress. Herein, we present different findings about the evolutionary biology of annual fish with emphasis on the genome dynamic and the population genomics linked to the speciation process in the genus *Austrolebias*.

## Living in Extreme Environments Displaying High Variability at Different Levels

Ponds in the temperate region of South America are heterogeneous environments that cover a large range of areas. They have variable depths, contain organic matter, are rich in invertebrates, plant diversity, and biomass, and are usually isolated from other ponds (Laufer et al., 2009; Arim et al., 2011). The fragmentary nature of this kind of habitat can induce the occurrence of evolutionary mechanisms such as genetic drift (Gillespie, 2000), bottlenecks, founder effects, and endogamy (Carvalho and Hauser, 1999) in annual fish. Long-term isolation among population also produces local differentiation driving to high levels of genetic and morphological diversity and endemisms in this South American region (García et al., 2002; Loureiro, 2004; Loureiro et al., 2018). However, previous genetic analyses have proposed that the *Austrolebias* population behave as a metapopulation. During the rainy season, floods mix the populations across large distances yielding a high intrapopulation genetic and morphological variation (García, 2006; García et al., 2009). The wide range of gene flow values revealed by analyses carried out with mitochondrial and nuclear markers indicated that it is not homogeneous among ponds (García et al., 2019, 2020). In fact, the estimates of gene flow reinforced the hypothesis that the populations under study live in a region with a complex geological history and in an irregular rainy regime yielding to chaotic local dynamics, which facilitate the persistence of such a metapopulation (García, 2006). On the other hand, this area has been affected by sea level fluctuations at least since the Miocene, between 15 and 30 million of years ago (Sprechmann, 1978), while the last transgression being five-thousand years ago (García-Rodriguez and Witkowaki, 2003). In this complex geomorphological scenario in the wetlands from South America the proposed explosive speciation events for the genus *Austrolebias* would be favored (García et al., 2002, 2014). The evolutionary history together with their peculiar development strategy and short generation time (1 year) could also favor high morphologic and molecular variability among and within ponds (García, 2006). Species size ranges from small (max. 35 mm of standard length) to large (more than 200 mm of standard length) (Huber, 1996; Loureiro and de Sá, 2016). Most large species are considered to form a monophyletic group of top predators (García et al., 2002; Costa, 2006, 2010). Moreover, striking sexual dimorphism has been reported for several species of rivulids fish and most Neotropical annual killifish. In *Austrolebias*, males are usually larger than females (Figures 1B–C). A study on intra- and intersexual selection revealed that a larger body size favors *A. charrua* males (Passos et al., 2013).

## Karytotypic Divergence Among Species of the Genus *Austrolebias*

In general, chromosome numbers vary little within and among teleost groups (Mank and Avise, 2006) and do not differ greatly from a widely proposed ancestral karyotype of 48 acrocentric chromosomes (Mank and Avise, 2006). This is also the case for some Neotropical rivulid species that include annual species (Elder et al., 1993; García et al., 1993, 1995, 2001). However, among annual killifish, the chromosome numbers range from 2N = 48 to 2N = 28 with arm numbers (NF) as high as 80 (García et al., 1993, 1995, 2001; García, 2016). In particular, chromosome variation at intra- and interspecific level was earlier described in species of the genus *Austrolebias* (García et al., 1993, 2001). Based on phylogenetic analysis using mitochondrial genes two major clades within *Austrolebias* could represent different repatterning pathways of the karyotypes (García et al., 2001; García, 2016). This analysis suggested that Clade I, corresponding to the *Austrolebias alexandri-affinis* species group, has differentiated basally during the late Miocene (García et al., 2014). This clade is characterized by a 2N = 48, different numbers of NF in each species, and the presence of extralarge acrocentric and subtelocentric chromosomes. Therefore, the occurrence of predominantly pericentric inversions, including perhaps heterochromatic loss/addition was proposed. On the other hand, Clade II is composed of all the remaining species groups clustered in four subclades showing the reduction of the diploid number from 2N = 48 to 2N = 34 and the maintenance of NF values nearly 48. Therefore, other rearrangement types could explain the chromosome evolution in these species groups (García et al., 1993, 2001).

## Giant Genomes and Speciation in the Neotropical Genus *Austrolebias*

Mank and Avise (2006) proposed that the genomes of ray-finned fishes (Actinopterygii) are well known for their evolutionary dynamism as reflected by drastic alterations in DNA content in general via partial or whole-genome duplication. Also, Mable et al. (2011) reported C-values for teleosts ranging between 0.35 and 4.9 pg, with an average of 1.2 pg. An amazing finding revealed that at least 16 species of *Austrolebias* show a C-value average of about 5.95 ± 0.45 pg/diploid cell (mean C-value of about 2.98 pg). In the same study, the genome size reported in the putative sister sympatric and syntopic taxon *C. melanotaenia* was of 2.72 ± 0.06 pg/diploid cell (García et al., 2014). Both C-values were corroborated in more recent findings through NGS of the total genome and *de novo* assembly and RNA-seq. This last analysis revealed haploid genomes size of 3.4 and 1.0 Gb for *A. charrua* and *C*. *melanotaenia* respectively (Valdivieso et al., 2017). The *C*. *melanotaenia* value fell within the range of most other cyprinodontoid and rivulid fish genomes, as in the early study in *Anablepsoides urophthalmus* (Hinegardner and Rosen, 1972), and more recently as in *Austrofundulus limnaeus* (Wagner et al., 2018), *Orestias ascotanensis* (Di Genova et al., 2022), and *Nematolebias whitei* (Thompson et al., 2022). Also, it is consistent with that of 3.11 pg/diploid cell reported in the African annual killifish *Nothobranchius furzeri* (Reichwald et al., 2009). Therefore, *Austrolebias* exhibit larger genomes compared to nearly all other reported diploid, i.e., non-(paleo) polyploid species of actinopterygian fishes (García et al., 2014). Previous analyses based on different mitochondrial genes supported the possible occurrence of burst cladogenetic processes in this genus. The sudden speciation in *Austrolebias* species, was preceded by events of divergence since a hypothetic ancestral rivulid genome which contained approximately 3.0 pg/diploid nucleus and a basal karyotype constituted by 48 small chromosomes of acrocentric type (García et al., 2001, 2002, 2014). These hypothesized events could be occurred since the Quaternary sharing drastic nuclear DNA increasing in all species analyzed, great genome instability and high levels of chromosomal divergence as previously mentioned (García et al., 2002; García, 2006; 2016). Moreover, they could explain the high morphological diversity described in species of the genus (Loureiro and de Sá, 1998; Loureiro et al., 2018).

## Massive Enrichment of Transposable Elements and Genome Instability as an Evolutionary Driving Force in *Austrolebias*

The extensive genetic variation by means of chromosome rearrangements involving both Robertsonian and non-Robertsonian changes at intra- and interspecific levels provides evidence for the genome instability occurring in *Austrolebias* (García et al., 1993, 2001). Remarkably, the frequency of these different types of chromosomal rearrangements between the two major clades occurred without statistically significant differences in nuclear DNA content. This finding reinforces the hypothesis that all *Austrolebias* species share similar nuclear DNA content since a hypothetic common ancestor (García, 2016).

Moreover, the drastic nuclear DNA–increasing events found in *Austrolebias* would be associated with considerable increase in the proportion of TEs. In fact, a comparative analysis of partial repetitive DNA content by means of NGS (New Generation Sequencing) revealed that the proportion of moderately repetitive DNA in *A. charrua* (45%) is approximately twice than that of the genus *C. melanotaenia* (25%) (García et al., 2015; Gutiérrez et al., 2016). Both species inhabit the same ponds and are distributed in the same South America temperate region. Most recently, total NGS genome analyses revealed a high number of repetitive elements present in *A. charrua* (68.1% of the total genome size), the most repetitive genome reported for a teleost (Valdivieso et al., 2017). Similar to the information reported by Chalopin et al. (2015) and Rhee et al. (2017), almost all classes of repetitive DNA are present in many teleosts groups and in the Rivulidae genomes. In the aforementioned comparative partial genome analysis implemented by García et al. (2015) in *A. charrua* and *C. melanotaenia,* retroelements make up most of the repetitive DNA. In particular, the *A. charrua* genome was predominantly enriched by LINE retroelements of the REX-Babar, Jockey, and L2 type (García et al., 2015; Valdivieso et al., 2017). Moreover, Rhee et al. (2017) reported that approximately one-fourth of the highly related mangrove killifish *Kryptolebias marmoratus* genome is composed of TEs, corroborating the previous finding in *C. melanotaenia*. Consistently, the most recent genome analysis of *O. ascotanensis* revealed that 21% (∼142 Mb) correspond to repetitive sequences, including LINE, LTR, and SINES sequences (Di Genova et al., 2022).

These results are in contrast to those reported in other related rivulid species as in the non-annual genome of mangrove killifish in which DNA transposons (approximately 10–14%) are relatively common (Rhee et al., 2017). TEs insertions can be responsible for the disruption of genes or regulatory sequences, and can also cause chromosomal rearrangements, representing a threat to their host genome integrity (Hedges and Deininger, 2007). The importance of TEs in the structure and evolution of vertebrate genomes and their major impact on genome diversity between and within lineages, were revisited by comparing the mobilomes of 23 vertebrate genomes (Chalopin et al., 2015). It was hypothesized that TE activation could promote or intensify morphological and karyotypical changes, some of which may be potentially important for the process of microevolution, and allow species with plastic genomes to survive as new forms or even as species in times of rapid climatic change (Belyayev, 2014). As proposed by this author, the scenario of events preceding speciation by different models in small marginal populations could be as follows: under the influence of unusual ecological conditions, TEs become active; the mobilization of TEs produces genetic variations, epigenetic alterations, and high rates of karyotypic change (including changes in species-specific chromosomal pattern). All these evolutionary hypothetic scenarios would be taken place in the genus *Austrolebias*.

Whereas the annotation and characterization of the complex genome sequence of the *A. charrua* and that of *C. melanotaenia* are in progress (Valdivieso et al., 2017), the comparative analysis of these genomes and mobilomes could help to interpret their possible association with the extensive phenotypic plasticity detected at all levels in species of the genus *Austrolebias*, adapted to extreme environmental conditions in temporary ponds.

## Additional Genomic Revolution and Phenotypic Innovations in Natural Hybrid Swarms of a Hybrid Zone Between Two *Austrolebias* Species

**FIGURE 2:**
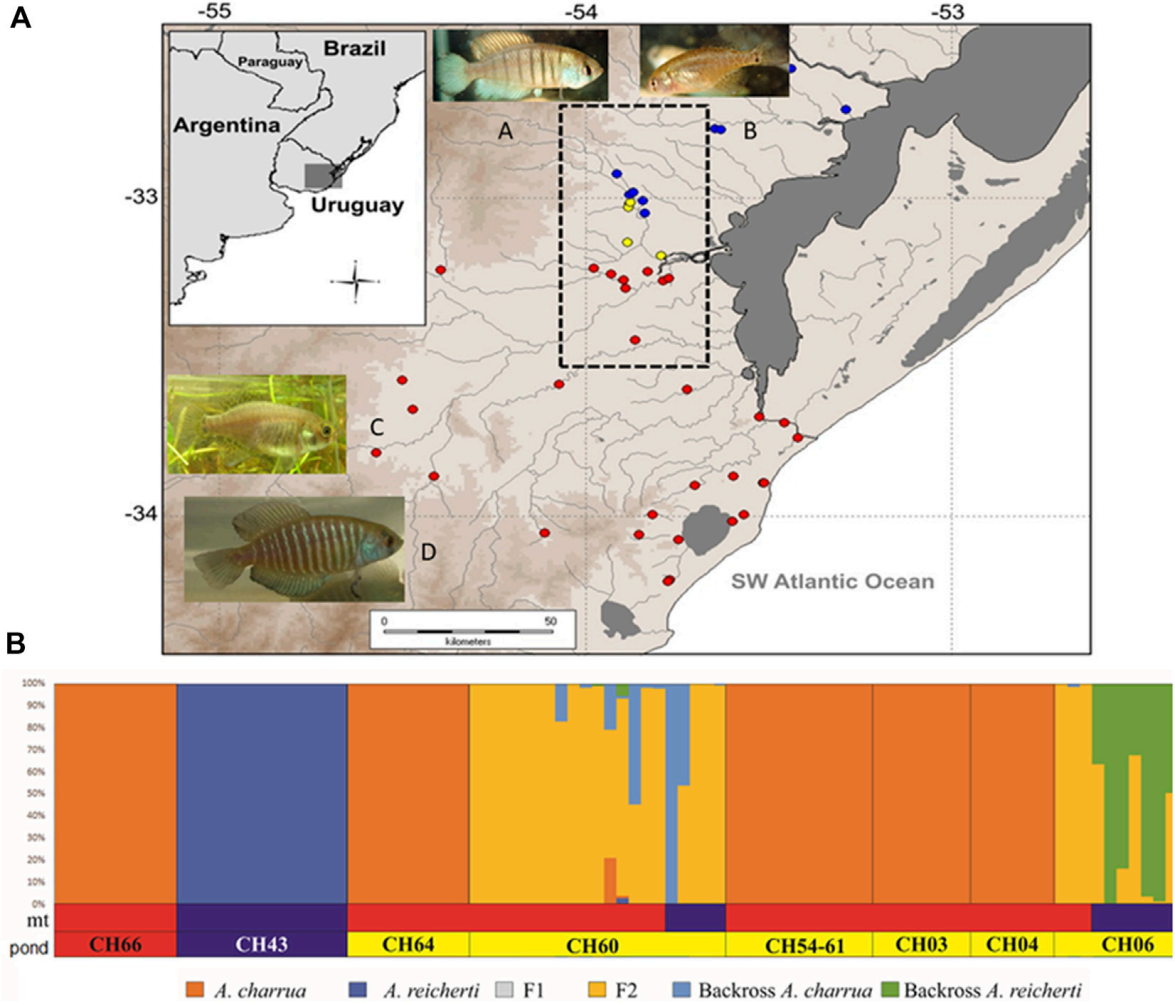
Geographic distribution and genotype assignment of individuals sampled in the hybrid zone of the Patos-Merin coastal lagoons basin system, South America. **(A)** The contact area between both taxa is delimited for the dotted line rectangle. Ponds are represented by circles: *A. charrua* (red circles), *A. reicherti* (blue circles) and hybrid populations (yellow circles) In the top, male **(A)** and female **(B)** of the parental species *A. reicherti*; in the bottom, female **(C)** and male **(D)** of the parental species *A. charrua*. **(B)** Posterior probabilities of the ancestral genotype class estimated with NewHybrids, under Uniform prior assumption. Each individual is represented as a vertical bar divided into six segments. Each color indicates the posterior probability of an individual assignment to pure *A. reicherti* (blue), pure *A. charrua* (red), F1 (grey), F2 (yelow), and first generation backcross of a F1 hybrid with a pure *A. reicherti* (BC1R, green) or with a pure *A. charrua* (BC1C, sky). Each of the sampled ponds in the hybrid zone are labeled below the bar plots, and named as follows: CH66, CH43, CH64, CH60, CH54-61, CH03, CH04, and CH06. mt (mitochondrial) bars: represent the Cytb haplotype assignation of each individual to *A. charrua* (red) or *A. reicherti* (blue) species. Modified from García et al. (2019).

Most recent population genetics analyses have detected other sources of morphological and genetic variability present in a contact area between two parapatric species *A. charrua* and *A. reicherti* (Figure 2A). This hybrid zone is the first described among Neotropical killifish and it was located in the Cebolattí River basin in Patos-Merin coastal lagoon system (García et al., 2019, 2020). The RNA-seq-based sequencing of the transcriptomes from pools of individuals of the two parental species and their putative natural hybrids allowed to identified a set of 111,725 SNP (single nucleotide polymorphism) markers, representing presumably fixed allelic differences among the two species (García et al., 2019). From these detected markers the first panel of 106 SNPs, in a single diagnostic multiplex assay to validate their capacity to reconstruct the patterns of the hybrid zone between both taxa, was performed. High-quality transcriptomes and a large set of gene-linked SNPs greatly facilitate functional and population genomics studies in the hybrid zone of these endangered species. Over the previously characterized bimodal hybrid zone (García et al., 2020) the new approach including more extensive sampling individuals and molecular markers, combined with morphological and biogeographic analyses, detected a population structure in which some groups among the hybrid swarms showed different level of introgression towards one or the other parental species according to their geographic distribution. In fact, after 14 years of fieldwork and laboratory analysis, the present hybrid zone remains localized and spatially reduced to two patches: 1) individuals showed intergradation of the morphological and pigmentation patterns towards *A. charrua*; 2) individuals morphologically *A. reicherti*-like. New Hybrid analysis (Figure 2B) suggested that the combination of hybrid genotypes and introgression generated new genomic entities, which are different from both parental taxa. Abundant empirical evidence shows that hybridization frequently leads to transgressive phenotypes in plants, animals, and fungi (Rieseberg et al., 1999; Langdon et al., 2019). Natural introgression between sympatric or parapatric sister species could be considered an *in situ* conservation strategy (Becker et al., 2013) and Hamilton and Miller (2015) have suggested that genetic variation that persists within natural hybrids may have conservation value. The present results reinforce the importance of the natural hybridization and introgression in the analyzed contact zone between *A. charrua* and *A. reicherti* to preserve *in situ* biodiversity by increasing the observed phenotypic variation in each temporal pond where these endangered killifish populations inhabit. Finally, hybridization between species could cause genomic stress, which can lead to several genome reorganizations that seem to be driven by TEs (Fontdevila, 2005; Michalak, 2009; Romero-Soriano et al., 2017). In this sense, earlier, Barbara McClintock (1984) proposed the genomic shock hypothesis in which the hybridization between two species constitutes a source of stress that could disrupt the control mechanisms of TEs and cause their activation.

## General Considerations and Open Questions

Among different genera of annual and non-annual killifish from the New and Old World, giant genomes (∼6 Gb) were only detected in *Austrolebias* (García et al., 2015; Valdivieso et al., 2017). Genome amplification has not occurred by polyploidization, since *Austrolebias* species are true diploids (García et al., 2014). Therefore, how can this formidable increase in genome size in the Neotropical genus *Autrolebias* be explained? Could it be related to the fact that *Austrolebias* is the unique rivulid genus that inhabit temperate Neotropical region? Could the extremely variable pond environments under different stress conditions have triggered unexpected TEs mobilization events? Are natural hybridization events between para/sympatric species linked to TEs mobilization in the genomes?

Previous findings in other taxa could contribute to our understanding of the possible underlying mechanisms to explain the existence of giant genomes and their dynamics in the genus *Austrolebias.* For example, the adaptation of *Drosophila* species to temperate climates was associated to widespread TEs (González et al., 2010). TEs invasion affected putatively genes which were also highly diverse in terms of their molecular and cellular function. Increasing the genome size as well as the timing of the development could have an impact also on its complexity (Gregory, 2005). Finally, an interesting and additional avenue to explore is the parallelism in the genomic size and the proportion of TEs (particularly, the abundance of LINE-type elements) between the human genome associated with different disorders and diseases and the *Austrolebias* one. For all the aforementioned issues, *Austrolebias* genus represents an outstanding species model in eco-evo-devo-research linking different evolutionary processes: drastic genome increase, massive presence of TEs, high chromosome instability, occurrence of natural hybridization between sister species, and the burst speciation process.
